# An airway epithelial IL-17A response signature identifies a steroid-unresponsive COPD patient subgroup

**DOI:** 10.1172/JCI121087

**Published:** 2018-11-26

**Authors:** Stephanie A. Christenson, Maarten van den Berge, Alen Faiz, Kai Inkamp, Nirav Bhakta, Luke R. Bonser, Lorna T. Zlock, Igor Z. Barjaktarevic, R. Graham Barr, Eugene R. Bleecker, Richard C. Boucher, Russell P. Bowler, Alejandro P. Comellas, Jeffrey L. Curtis, MeiLan K. Han, Nadia N. Hansel, Pieter S. Hiemstra, Robert J. Kaner, Jerry A. Krishnanm, Fernando J. Martinez, Wanda K. O’Neal, Robert Paine, Wim Timens, J. Michael Wells, Avrum Spira, David J. Erle, Prescott G. Woodruff

**Affiliations:** 1 Department of Medicine, UCSF, San Francisco, California, USA.; 2 University Medical Center Groningen, Department of Pulmonary Diseases and Research Institute for Asthma and COPD (GRIAC), Groningen, Netherlands.; 3 Department of Pathology, UCSF, San Francisco, California, USA.; 4 Department of Medicine, UCLA, Los Angeles, California, USA.; 5 Department of Medicine, Columbia University Medical Center, New York, New York, USA.; 6 Department of Medicine, University of Arizona, Tucson, Arizona, USA.; 7 Marsico Lung Institute, University of North Carolina at Chapel Hill, Chapel Hill, North Carolina, USA.; 8 National Jewish Health, Denver, Colorado, USA.; 9 Department of Medicine, University of Iowa, Iowa City, Iowa, USA.; 10 Department of Medicine, University of Michigan, Ann Arbor, Michigan, USA.; 11 Department of Medicine, Johns Hopkins University, Baltimore, Maryland, USA.; 12 Department of Pulmonology, University Medical Center, Leiden, Netherlands.; 13 Department of Medicine, Weill Cornell Medical Center, New York, New York, USA.; 14 Breathe Chicago Center, University of Illinois at Chicago, Chicago, Illinois, USA.; 15 Department of Internal Medicine, University of Utah, Salt Lake City, Utah, USA.; 16 University Medical Center Groningen, Department of Pathology and Medical Biology and Research Institute for Asthma and COPD (GRIAC), Groningen, Netherlands.; 17 Department of Medicine, University of Alabama at Birmingham, Birmingham, Alabama, USA.; 18 Department of Medicine, Boston University School of Medicine, Boston, Massachusetts, USA.

**Keywords:** Immunology, Pulmonology, Adaptive immunity, Bioinformatics, COPD

## Abstract

**BACKGROUND.** Chronic obstructive pulmonary disease (COPD) is a heterogeneous smoking-related disease characterized by airway obstruction and inflammation. This inflammation may persist even after smoking cessation and responds variably to corticosteroids. Personalizing treatment to biologically similar “molecular phenotypes” may improve therapeutic efficacy in COPD. IL-17A is involved in neutrophilic inflammation and corticosteroid resistance, and thus may be particularly important in a COPD molecular phenotype.

**METHODS.** We generated a gene expression signature of IL-17A response in bronchial airway epithelial brushings from smokers with and without COPD (**n** = 238), and validated it using data from 2 randomized trials of IL-17 blockade in psoriasis. This IL-17 signature was related to clinical and pathologic characteristics in 2 additional human studies of COPD: (a) SPIROMICS (**n** = 47), which included former and current smokers with COPD, and (b) GLUCOLD (**n** = 79), in which COPD participants were randomized to placebo or corticosteroids.

**RESULTS.** The IL-17 signature was associated with an inflammatory profile characteristic of an IL-17 response, including increased airway neutrophils and macrophages. In SPIROMICS the signature was associated with increased airway obstruction and functional small airways disease on quantitative chest CT. In GLUCOLD the signature was associated with decreased response to corticosteroids, irrespective of airway eosinophilic or type 2 inflammation.

**CONCLUSION.** These data suggest that a gene signature of IL-17 airway epithelial response distinguishes a biologically, radiographically, and clinically distinct COPD subgroup that may benefit from personalized therapy.

**TRIAL REGISTRATION.** ClinicalTrials.gov NCT01969344.

**FUNDING.** Primary support from the NIH, grants K23HL123778, K12HL11999, U19AI077439, DK072517, U01HL137880, K24HL137013 and R01HL121774 and contracts HHSN268200900013C, HHSN268200900014C, HHSN268200900015C, HHSN268200900016C, HHSN268200900017C, HHSN268200900018C, HHSN268200900019C and HHSN268200900020C.

## Introduction

Personalizing treatment to “molecular phenotypes,” i.e., to subsets of patients with shared underlying biology, is an emerging strategy to guide therapeutic choices in chronic disease (1, 2). In respiratory disease, this strategy has particularly gained traction in severe asthma, where subgroups of patients with type 2 and eosinophilic inflammation can be targeted using new biologics (1–4). Much of the inflammation in chronic respiratory disorders, however, does not respond to therapies directed against type 2 inflammation. Identifying subgroups that display enhanced non–type 2 inflammatory pathways may lead to the repurposing of available biologics indicated for other inflammatory disorders to target these subgroups.

Chronic obstructive pulmonary disease (COPD) is a highly prevalent respiratory disease, most commonly associated with smoking. COPD is a major cause of morbidity and mortality worldwide for which few interventions have been found that prevent disease progression (5). Yet molecular phenotyping has been less studied in COPD than in asthma and has focused on eosinophilic and type 2 inflammation based on the previous work in asthma (2). Type 2 inflammation is likely relevant in only a minority of COPD patients (6). Nonetheless, this work suggests that biologically distinct COPD subgroups exist and are clinically relevant. COPD patients with high eosinophil counts or an airway epithelial genomic signature of type 2 inflammation are more likely to respond to corticosteroids, and potentially to biologics targeting eosinophils (6–9). These studies suggest the promise of molecular phenotyping in COPD, but responses beyond type 2 inflammation require further investigation.

The IL-17 family of cytokines includes 6 members that play various roles in mucosal host defense and chronic inflammation (10). IL-17A stimulates the airway epithelium to produce chemokines and other mediators, which recruit and activate neutrophils and macrophages, cells crucial to COPD pathogenesis (11). IL-17A is implicated in COPD-associated pathogenic responses, including emphysema, lymphoid neogenesis, corticosteroid resistance, dysbiosis, mucus hypersecretion, and ongoing inflammation despite smoking cessation (12–19). However, many of these responses have not been investigated in human studies. By identifying the COPD subgroup that manifests IL-17A–associated inflammation (IL-17A is hereafter referred to as IL-17), we hypothesize that we can distinguish a corticosteroid-unresponsive subgroup that may benefit from anti–IL-17 biologics. Anti–IL-17 biologics are now approved for the treatment of autoimmune diseases, specifically psoriasis and psoriatic arthritis, and are being studied in COPD (20, 21). Nontargeted trials of biologic therapies in COPD have failed to meet clinical endpoints, suggesting the importance of directing therapy to appropriate subgroups (22).

Here we studied the transcriptional response of the airway epithelium to IL-17. We have found that direct measurement of interleukin proteins, including IL-17, can be difficult in human blood and bronchoalveolar lavage fluid (23, 24). These challenges may contribute to the inconsistent evidence for IL-17 protein levels being increased in COPD (25–30). Conversely, airway epithelial cells have reproducible transcriptional responses to many interleukins. Thus our general strategy has been to assay this epithelial response, which we have validated for IL-13 (24, 31, 32) and interferons (33).

**Figure 1:**
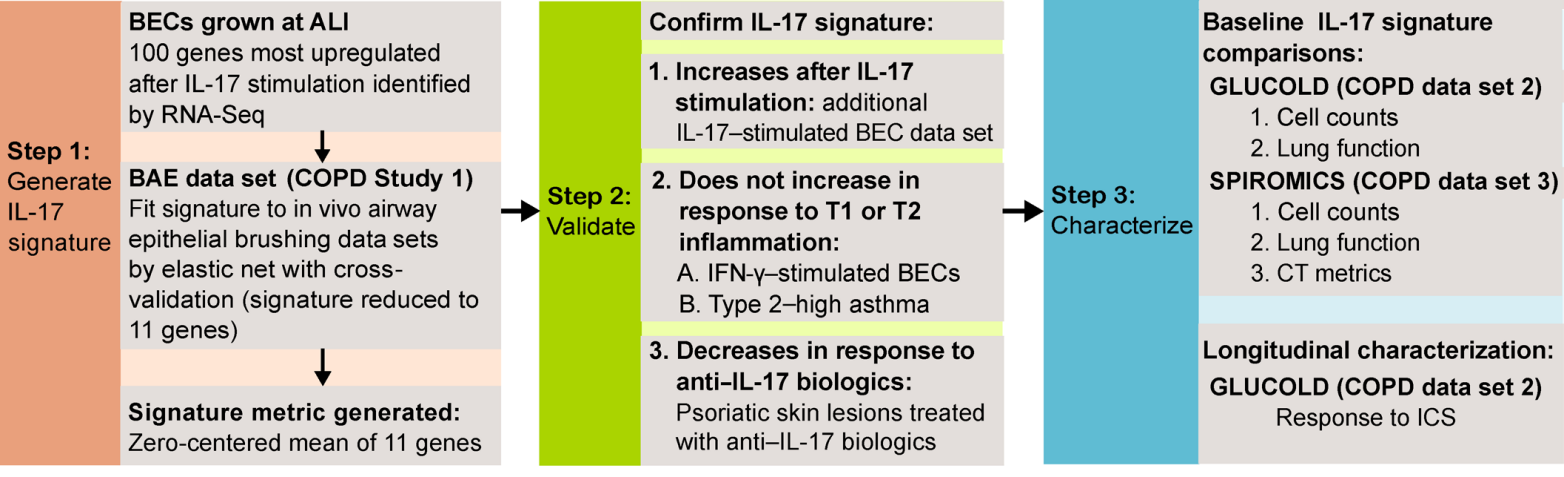
Study design. ALI, air-liquid interface; BAE, bronchial airway epithelial; BEC, bronchial epithelial cell; ICS, inhaled corticosteroid; RNA-Seq, RNA sequencing; T1, type 1 inflammation; T2, type 2 inflammation.

We examined a genomic signature of the airway epithelial IL-17 response in 3 separate human COPD studies in which bronchial airway samples were collected during bronchoscopy. We first fit the IL-17 genomic signature, generated using bronchial epithelial cells exposed to IL-17, to a cross-sectional study of ever-smokers with and without COPD (bronchial airway epithelial [BAE] data set, *n* = 237). We next established that the signature specifically identified IL-17–associated inflammation by determining its response to other airway epithelial adaptive immune responses (types 1 and 2) and to IL-17–directed biologic therapies in psoriatic skin lesions. We then tested the associations between this IL-17 signature and clinical features in 2 independent COPD studies that collected rich phenotypic data (GLUCOLD, *n* = 79, and SPIROMICS, *n* = 47; study design in Figure 1). We hypothesized that our airway epithelial IL-17 genomic signature would be increased in a COPD subset, and associated with distinct clinical, pathologic, and radiographic characteristics.

## Results

### Generation of an airway epithelial IL-17–associated gene expression signature in COPD.

We first characterized the airway epithelial response to IL-17 using whole transcriptome profiling of IL-17–stimulated versus matched unstimulated control human bronchial epithelial cell (HBEC) cultures grown at air-liquid interface (ALI). The 100 genes most upregulated by log_2_ fold change in response to IL-17 were studied as candidate IL-17 signature genes.

We examined these 100 genes in a previously generated BAE transcriptome profiling data set derived from bronchoscopic brushing samples from ever-smokers with (*n* = 85) and without COPD (*n* = 152; BAE data set; demographics in Table 1). Candidate IL-17 signature genes were enriched among smokers with COPD compared with those without (mean of the zero-centered log_2_ gene expression in those with COPD = 0.11 ± 0.27 vs. without = –0.60 ± 0.19, *P* = 6.10 × 10^–6^; Supplemental Figure 1; supplemental material available online with this article; https://doi.org/10.1172/JCI121087DS1).

**Figure 2:**
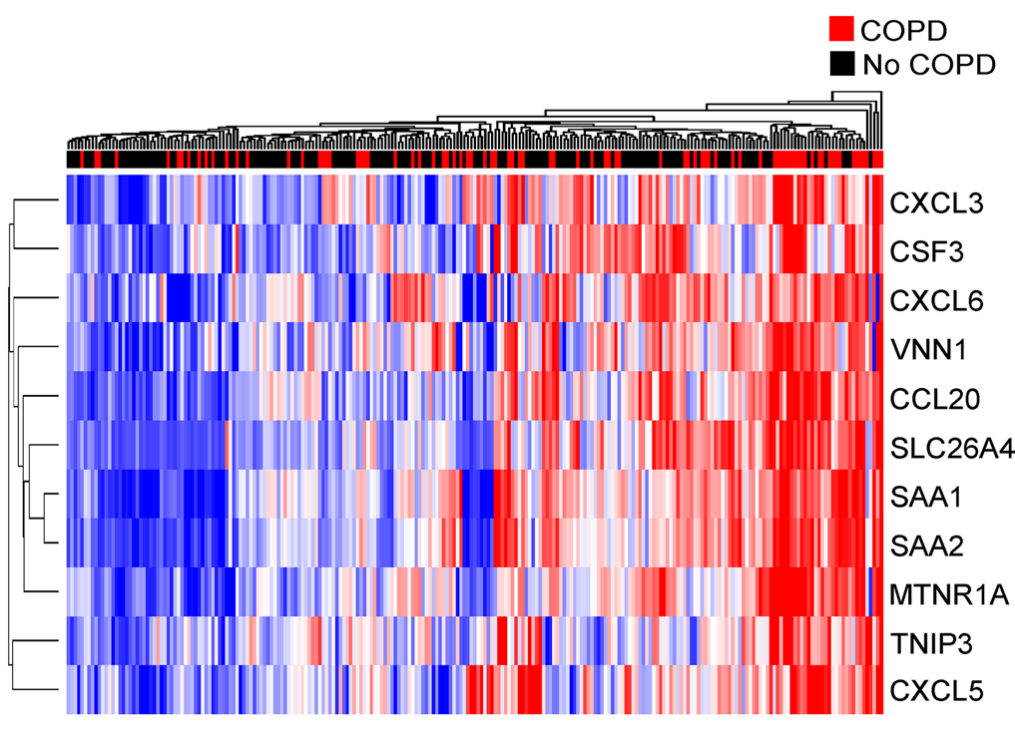
Hierarchical clustering of the 11 IL-17 signature genes in the BAE data set (*n* = 237). Signature genes are shown in rows across participants in columns. Blue and red indicate low and high relative gene expression, respectively. Smokers with and without COPD are indicated by red and black, respectively, in the above color bar. Clustering across participants and genes was done by Euclidean distance with average linkage.

Next, we generated a genomic signature of the IL-17 response specific to in vivo brushing samples from smokers by restricting the 100-gene signature identified in the culture model to those tightly correlated in the BAE data set using an elastic net (34, 35). We took this additional step because cell culture models cannot optimally reproduce the complexity of the in vivo environment in which multiple mechanistic pathways impact gene expression, often with disparate effect. This signature refinement process is based on the premise that highly intercorrelated genes are coregulated by the same molecular processes, a premise also used by pathway analysis tools such as weighted gene coexpression analysis (36, 37). Starting with the 100 candidate genes as predictors, elastic net regression with leave-one-out cross-validation selected 10 genes highly correlated with a representative IL-17–related gene, *CCL20,* in the BAE data set (Figure 2). We chose *CCL20* a priori to guide the elastic net gene selection to specifically identify an IL-17/*CCL20–*associated response. *CCL20* was chosen for this role based on (a) biological relevance, as an epithelial gene known to be the only ligand for CCR6, a chemokine receptor preferentially expressed by Th17 cells, and thus thought to be more specific for an IL-17 response as compared with other adaptive immune responses (38); and (b) statistical relevance, because it was highly upregulated (log_2_ fold change = 2.92, FDR = 0.0006) following IL-17 stimulation in vitro. Importantly, this IL-17–associated gene was chosen to guide gene selection because our goal was to retain co-associated genes given their potential biological relevance, independent of outcomes of interest. We confirmed that the 10 genes selected by elastic net and *CCL20* were all intercorrelated (Supplemental Figure 2), verifying that the elastic net procedure removed loosely correlated genes. Nearly all of the 10 genes have previously been shown to be associated with IL-17–related inflammation (38–42). We thus used these 10 genes, along with *CCL20*, to construct a gene signature of airway epithelial response to IL-17 using the mean of their zero-centered log_2_ expression values.

We confirmed that the genes selected for the signature were measuring an IL-17 response not just specific to *CCL20* in two ways. First, we evaluated the correlation between our IL-17 signature and a 5-gene airway epithelial IL-17 gene expression metric previously examined in asthma (39). In the BAE data set the 2 signatures were well correlated (ρ = 0.51, *P* < 2.2 × 10^–16^) in COPD participants (Supplemental Figure 3 and Supplemental Table 1). The signatures were also correlated in an additional COPD data set, GLUCOLD (demographics in Table 1), in which transcriptomic profiles from endobronchial biopsies were obtained from 79 participants with COPD (ρ = 0.49, *P* = 5.0 × 10^–6^; Supplemental Figure 3 and Supplemental Table 1). Second, we repeated the elastic net procedure using *SLC26A4*, the gene most upregulated with IL-17 stimulation in cell culture also measured in the COPD array data (log_2_ fold change = 8.51, FDR = 0), to guide the elastic net. The *SLC26A4*-based signature incorporated 16 genes, 6 of which were also in the 11-gene *CCL20*-based signature, and was highly correlated with the *CCL20*-based signature in the BAE and GLUCOLD data sets (ρ = 0.97 and *P* < 2.2 × 10^–16^, ρ = 0.87 and *P* < 2.2 × 10^–16^, respectively; Supplemental Figure 4 and Supplemental Table 1). Thus, removal of loosely associated genes from the 100-gene signature using *CCL20* to guide the elastic net measured a response that does not appear to be exclusive to *CCL20*. However, we used the *CCL20*-based signature for our subsequent analyses, as it had clear advantages over the others. The asthma signature was generated in a cell culture model and never fit to the in vivo environment. *SLC26A4* is of unclear significance in IL-17 biology, and thus we considered the *CCL20*-based signature more biologically relevant.

### IL-17–related gene expression confirmed in an additional airway epithelial culture data set.

We validated the association between the 10 genes selected by elastic net and IL-17 stimulation in another publicly available microarray data set of HBECs grown at ALI and stimulated with IL-17 for 24 hours (as opposed to the 7-day stimulation in our culture model) (Gene Expression Omnibus [GEO] GSE10240) (43). Although 2 of the 10 genes (*SAA1* and *SAA2*) were poorly annotated on this array and could not be measured, the rest were significantly upregulated after IL-17 stimulation in this validation data set (7 of 8 were within the top 50 genes by log_2_ fold change) despite differences in cytokine stimulation time.

### IL-17–related gene expression measures a response distinct from type 1 and 2 immune responses.

Only 3 of the 11 IL-17 signature genes were significantly altered after HBECs at ALI were stimulated with IFN-γ, the main cytokine released from Th1 and Tc1 cells, and thus indicative of a type 1 response. Two of the genes were repressed and one induced with an overall mean log_2_ fold change of –0.19 (Supplemental Table 2). None of the genes were significantly upregulated in steroid-naive mild to moderate asthmatics previously shown to have high type 2 gene expression (*n* = 40) compared with asthmatics with low type 2 expression (*n* = 22) and healthy controls (*n* = 43) (Supplemental Table 3).

### Decreased IL-17 signature expression following IL-17 blockade in psoriatic lesions.

To further validate that our IL-17 signature reflects an IL-17 response, we examined it in 2 publicly available transcriptomic data sets of psoriatic skin lesions before and after controlled treatment with anti–IL-17 biologics.

**Figure 3:**
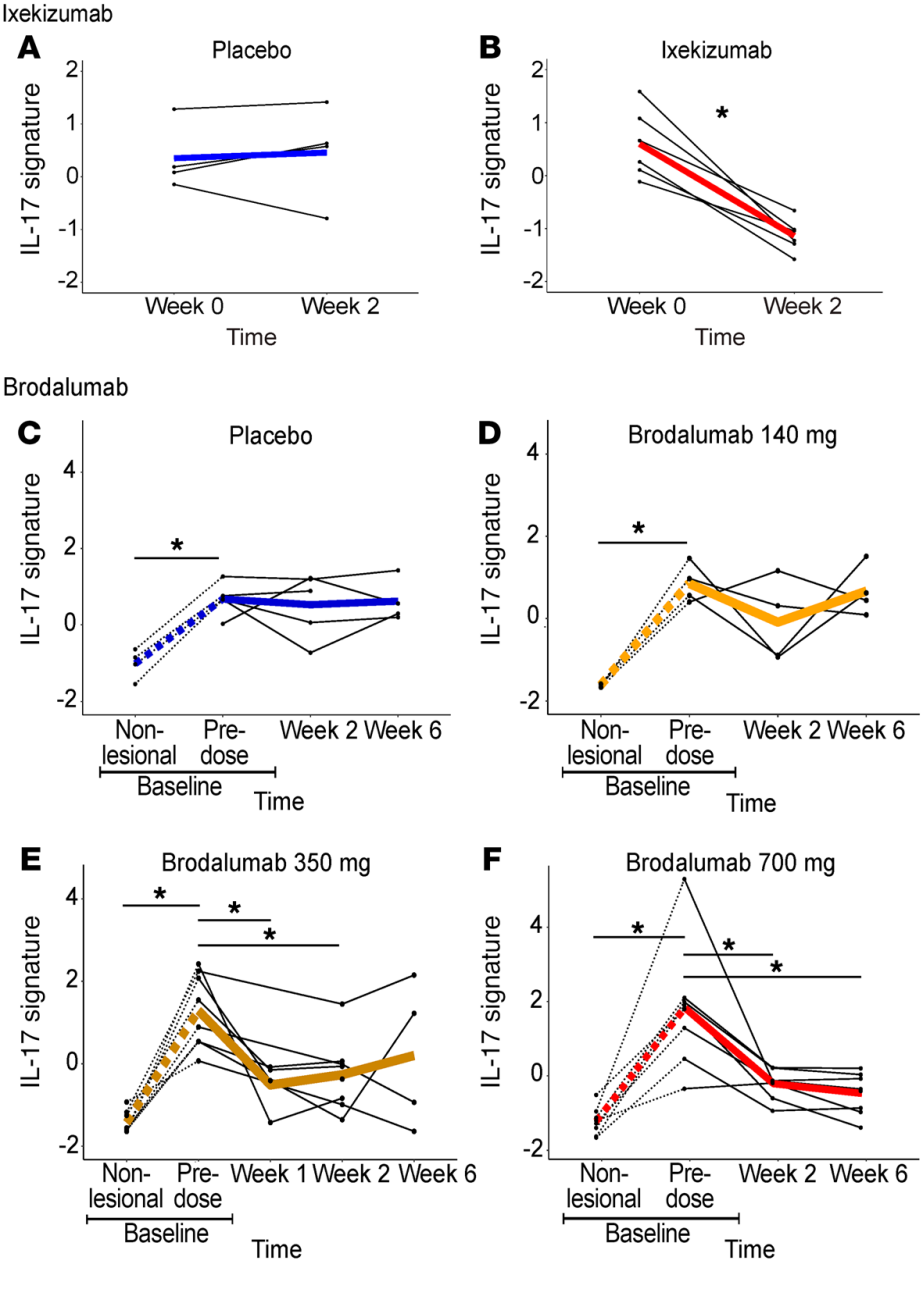
IL-17 blockade in psoriasis. Ixekizumab: compared with placebo (**A**) (*n* = 4), the IL-17 signature was decreased in psoriatic lesions (*n* = 6) after 2 weeks of ixekizumab (**B**). (**C**–**F**) Brodalumab: Compared with placebo (**C**) (*n* = 5), brodalumab (*n* = 20) at a dose of 140 mg did not (**D**), but at a dose of 350 mg (at 1 and 2 weeks) (**E**) or 700 mg (at 2 and 6 weeks) (**F**) did, result in a decrease in the IL-17 signature, consistent with clinical response. The IL-17 signature was higher in psoriatic lesions than in matched nonlesional skin samples (**C**–**F**, dashed lines). *P < 0.05 using mixed-effects models.

In the first data set (GEO GSE31652) (44), psoriatic skin lesion biopsies were taken at baseline and after 2 weeks of ixekizumab, an anti–IL-17 monoclonal antibody (*n* = 6), or placebo (*n* = 4). All ixekizumab-treated participants, but none of the placebo-treated, showed clinical improvement of at least 75% at 6 weeks. The skin IL-17 gene signature decreased over 2 weeks in lesions from ixekizumab- but not placebo-treated participants (*P* = 0.003 for the interaction between treatment and time; Figure 3, A and B).

In the second data set (GEO GSE53552) (45), biopsies were taken from psoriatic skin lesions and matched nonlesional skin at baseline (*n* = 25). Psoriatic lesions were then sampled over 6 weeks after treatment with placebo (*n* = 5) or a dose range of brodalumab (*n* = 20), an IL-17 receptor-α–blocking monoclonal antibody. Psoriatic lesions showed higher IL-17 signature expression compared with matched nonlesional skin (*P* = 0.001; Figure 3, C–F). The signature decreased over time in psoriatic lesions in those who received 350 or 700 mg compared with placebo, but not in those who received 140 mg (350 mg: *P* = 0.005 at 1 week, *P* = 0.02 at 2 weeks, and *P* = 0.12 at 6 weeks; 700 mg: *P* = 0.002 at 2 weeks and 0.0006 at 6 weeks for the interaction between treatment and time; Figure 3, C–F). This was consistent with clinical treatment response (all placebo-treated and three of four 140-mg-treated participants showed no clinical treatment response; all 700-mg-treated and all but one 350-mg-treated showed at least 70% clinical improvement). The observation that our putative IL-17 signature tracked with clinical response to an IL-17 inhibitor in 2 psoriasis clinical trials provides independent confirmation of its value as a metric of IL-17–driven inflammation.

### Cross-sectional characterization of an IL-17 gene signature in the BAE data set.

**Figure 4:**
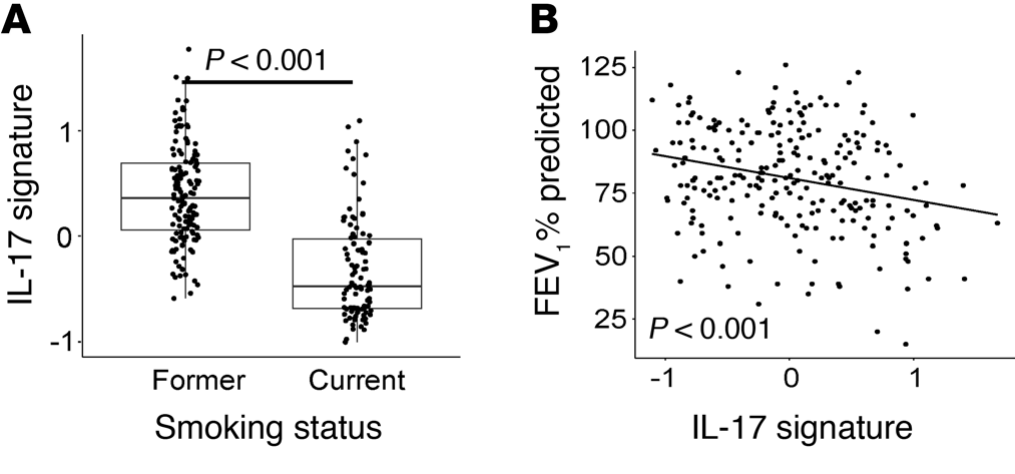
Clinical correlations in the BAE dataset. The IL-17 gene signature in the BAE data set (*n* = 237) was increased in former (0.29 ± 0.46) compared with current smokers (–0.42 ± 0.48, *P* < 0.001 by Wilcoxon rank sum test) (**A**), and associated with decreasing FEV_1_% predicted (ρ = –0.23, *P* < 0.001 by Spearman’s correlation) (**B**).

In the BAE data set, our 11-gene IL-17 signature was higher in former smokers (mean of the zero-centered log_2_ gene expression = 0.29 ± 0.46) compared with current smokers (–0.42 ± 0.48, *P* < 2.2 × 10^–16^; Figure 4A and Supplemental Table 4), and was associated with older age (ρ = 0.19, *P* = 0.004). The signature was increased in COPD compared with ever-smokers without COPD (i.e., those with preserved lung function; 0.21 ± 0.66 and –0.12 ± 0.51, respectively, *P* = 1.34 × 10^–5^), even after adjustment for smoking status and age (*P* = 6.2 × 10^–6^). The signature was also higher with decreasing lung function (defined as the volume of air exhaled in the first second of a forced expiratory maneuver [FEV_1_]). Specifically, a higher gene signature was associated with lower FEV_1_ expressed as a percentage of the predicted value (FEV_1_% predicted) across all participants (1 unit increase in the IL-17 signature is associated with a 12-ml decrease in FEV_1_, *P* = 1.40 × 10^–5^) and among only COPD participants (associated with a 5.5-ml decrease in FEV_1_, *P* = 0.04), suggesting an association with increasing COPD severity (Figure 4B).

### Cross-sectional characterization in GLUCOLD and SPIROMICS.

We next studied baseline clinical characteristics associated with the IL-17 signature in GLUCOLD and another COPD data set, SPIROMICS (demographics in Table 1). GLUCOLD included endobronchial biopsy transcriptomic profiles from steroid-naive participants with moderate to severe COPD (*n* = 79). SPIROMICS included bronchial epithelial brushing profiles from ever-smokers with mild to moderate COPD (*n* = 47). As in the BAE data set, in both GLUCOLD and SPIROMICS the IL-17 signature was associated with increasing age (ρ = 0.24, *P* = 0.039, and ρ = 0.20, *P* = 0.046, respectively) and was higher in former compared with current smokers (*P* = 2.42 × 10^–6^ and 1.35 × 10^–5^, respectively; Supplemental Table 4). We performed subsequent analyses before and after adjustment for age and smoking status.

### Association with increased airway neutrophils and macrophages.

**Figure 5:**
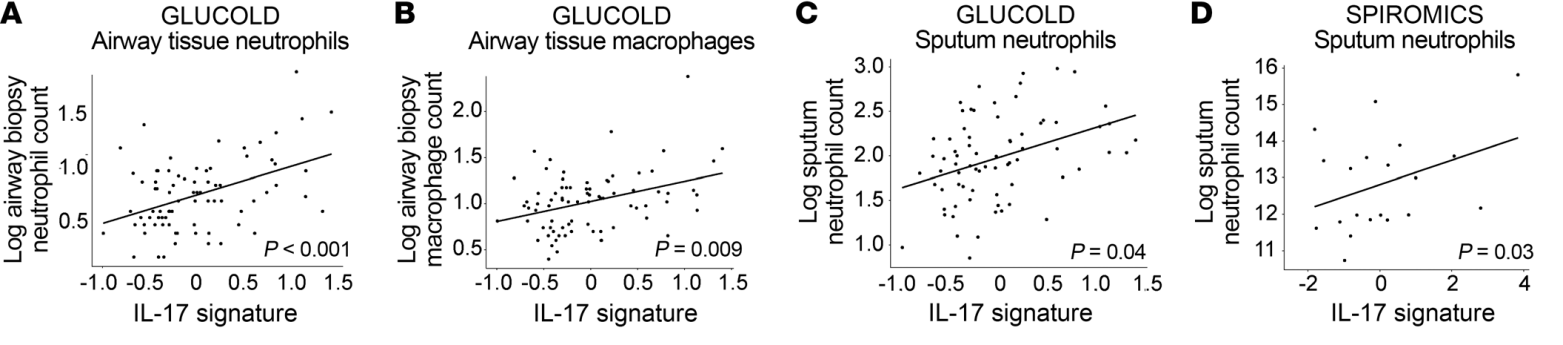
Airway neutrophils and macrophages. (**A**–**C**) GLUCOLD (*n* = 79): The IL-17 signature was associated with increasing log_2_ counts of airway tissue neutrophils (**A**), airway tissue macrophages (**B**), and sputum neutrophils (**C**) (*n* = 72 with measured neutrophils). (**D**) SPIROMICS: The signature was associated with log_2_ sputum neutrophil counts (*n* = 20). *P* values shown for linear models adjusted for age and smoking status.

In GLUCOLD, the IL-17 signature was associated with increased airway biopsy neutrophil (*P* = 6.41 × 10^–5^; Figure 5A) and macrophage counts (*P* = 0.009; Figure 5B), but not eosinophils, mast cell counts, or our previously described type 2 genomic signature score (TS2) (Table 2). Tissue cell counts and the T2S score were not measured in SPIROMICS, but the T2S score was also not associated with the IL-17 signature in the BAE data set (Table 2). The IL-17 signature was moderately associated with sputum neutrophil counts in both GLUCOLD (*P* = 0.041; Figure 5C) and SPIROMICS (*P* = 0.033; Figure 5D), although this did not stand up to multiple-comparisons adjustment. There was no association with sputum eosinophil counts or any blood cell counts (Table 2).

### Association with airway obstruction.

As in the BAE data set, in SPIROMICS we found that a higher IL-17 signature was associated with slightly greater airway obstruction in COPD (*P* = 0.038 after adjustment for smoking and age; Supplemental Figure 5 and Table 3), although this was not significant after adjustment for multiple comparisons. In GLUCOLD we found a trend toward an association (*P* = 0.06 before and *P* = 0.12 after adjustment for smoking and age; Supplemental Figure 5 and Table 3).

### Association with CT measurements of functional small airways disease.

**Figure 6:**
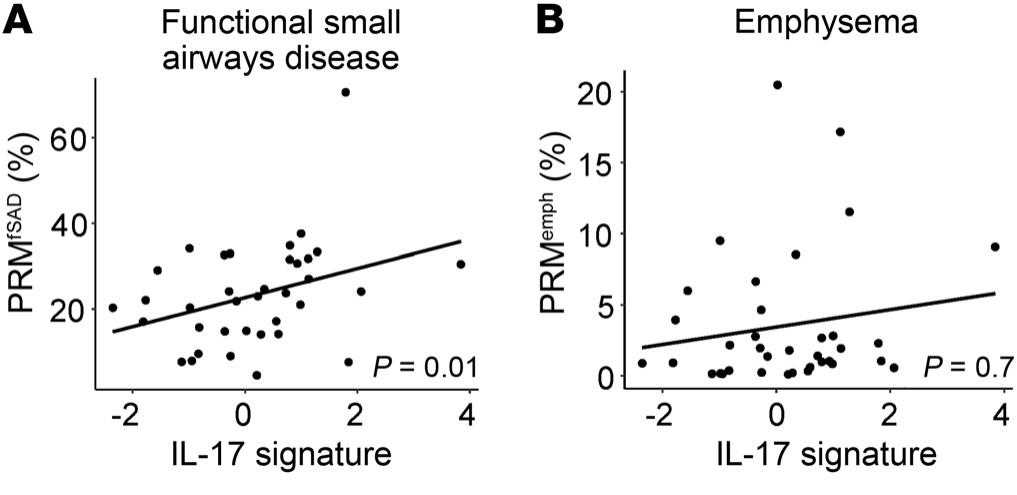
CT biomarkers. The IL-17 signature was associated with increasing percentage of lung area with functional small airways disease (PRM^fSAD^) (**A**) but not emphysema (PRM^emph^) (**B**) by parametric response mapping of baseline CT scans (*n* = 35). *P* values shown for linear models adjusted for age and smoking status.

In SPIROMICS, we obtained inspiratory and expiratory quantitative chest CT scans at study entry. We found that the IL-17 signature was associated with an increase in air trapping in areas devoid of emphysema (known as functional small airways disease) by parametric response mapping (PRM) analysis (PRM^fSAD^) (*P* = 0.01; Figure 6A, Table 3, and ref. 46). The IL-17 signature was not associated with PRM-measured emphysema (PRM^emph^). However, almost all participants who underwent bronchoscopy had mild disease, with very few displaying significant emphysema (Figure 6B).

### Association with decreased response to inhaled corticosteroids in GLUCOLD.

**Figure 7:**
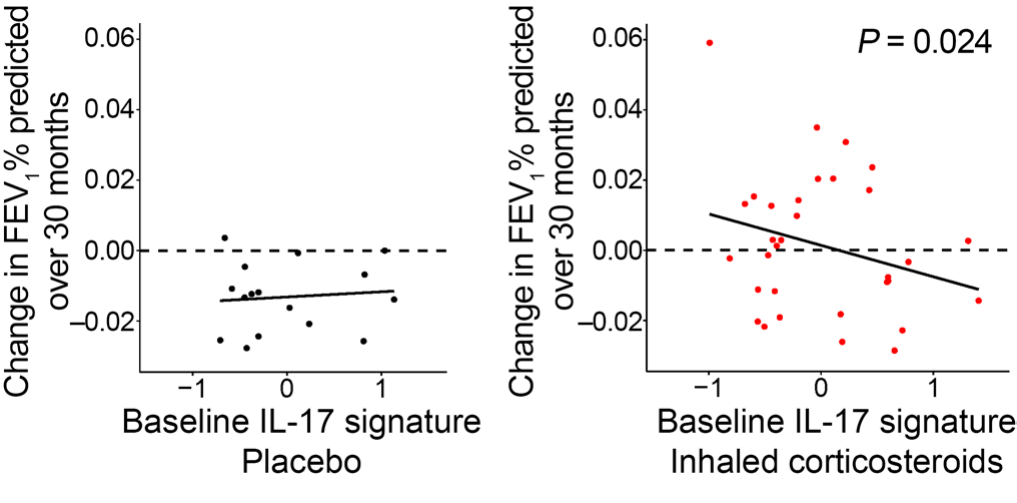
ICS response in GLUCOLD. An increased baseline IL-17 signature was associated with a greater decrease in percentage change in FEV_1_ in the ICS ± long-acting β-agonist group (*n* = 33) compared with placebo (*n* = 16) at 30 months (*P* = 0.024 for the linear model interaction with adjustment for age and smoking status). Participants with low IL-17 signatures were more likely to show an improvement in FEV_1_ after ICS (greater than zero: above the dashed line).

Following baseline bronchoscopy in GLUCOLD, 49 participants with available baseline biopsies were randomized to treatment with 30 months of medication containing inhaled corticosteroid (ICS) (*n* = 33) or placebo (*n* = 16). A higher baseline IL-17 signature was associated with lack of improvement in post-bronchodilator FEV_1_ on ICS, whereas a lower IL-17 signature was associated with improvement in FEV_1_, as compared with placebo (*P* = 0.028 for the interaction between treatment and time; Figure 7 and Table 3). We identified 28% of GLUCOLD participants as having high IL-17 gene expression (“IL-17–high”) by cluster partitioning (31% of COPD participants over all 3 studies, including 33% of BAE and 34% of SPIROMICS participants, were IL-17 high; Supplemental Figure 6). After categorization of participants based on this cluster partitioning, those with an “IL-17–low” designation were more likely to respond to ICS with an improvement in lung function, while IL-17 high was associated with lack of response to ICS at 30 months (*P* = 0.047 for the interaction between IL-17 status and percentage change in FEV_1_ after ICS compared with placebo).

We found that a high IL-17 signature was specific but not sensitive for steroid unresponsiveness. Using the dichotimization into IL-17 high and low by cluster partitioning, the specificity for steroid unresponsiveness was 75% (Supplemental Table 5). When the IL-17–high group was restricted to a slightly higher cutoff at the top quartile of IL-17 signature values, the specificity increased to 94% (Supplemental Table 6).

The association between the IL-17 signature and change in FEV_1_ among ICS-treated participants was not due to those participants with low IL-17 signature expression reciprocally exhibiting high type 2 inflammation. The significance of the relationship between the IL-17 signature and ICS response persisted even after we adjusted for markers of steroid-responsive type 2 inflammation using either airway tissue eosinophils (*P* = 0.027) or our previously identified airway epithelial genomic signature of type 2 inflammation (*P* = 0.018; Supplemental Figure 7, Table 3, and ref. 6). The association also does not appear to be explained by IL-17 inflammation simply reflecting tissue neutrophils or macrophages, as adjustment for neutrophil or macrophage counts in the model also did not change the relationship between the IL-17 signature and ICS response (*P* = 0.016 and 0.030, respectively; Table 3).

The IL-17 signature alone explained 23% of the variation in change in FEV_1_ with corticosteroids (*r*^2^ = 0.23; Table 3). As expected given the low sensitivity of the IL-17 signature for steroid unresponsiveness, the area under the receiver operating characteristic curve (AUC) was modest (63%; Supplemental Figure 8). However, there were no significant associations between other biomarkers of inflammation (including sputum and blood cell counts) and change in FEV_1_ in ICS- versus placebo-treated participants after adjustment for age and smoking status. Furthermore, the AUCs for these other potential biomarkers (sputum eosinophils, 51%; blood eosinophils, 55%; sputum neutrophils, 52%; blood neutrophils, 45%) suggest that they lack any predictive power for corticosteroid responsiveness in this data set (Supplemental Figure 8). Although limited by small sample size, these proof-of-concept analyses suggest that our airway epithelial signature of IL-17 response in COPD may mark FEV_1_ response to ICS better than easily measured cell differentials or other genomic markers of the adaptive immune response.

## Discussion

In this study, we used 3 complementary human COPD studies to characterize the clinical significance of the airway epithelial response to IL-17 in COPD. We showed that a signature of IL-17–associated airway inflammation is upregulated in a subset of participants with COPD (31% across studies), and is associated with distinct inflammatory, physiologic, and clinical features. Increases in this signature are associated with an inflammatory profile characteristic of an IL-17 response, including increased airway neutrophils and macrophages but not eosinophils, type 2 markers, or type 1 gene expression. Decreases in the signature occur in response to therapeutic blockade of IL-17 in psoriatic skin lesions, and this response corresponds to clinical improvement in that disease. In COPD, the signature is further associated with more severe airway obstruction and a novel CT biomarker of functional small airways disease that is predictive of worsening airway disease over time (46, 47). Finally, higher IL-17 signature expression is associated with a lack of response to ICS in COPD, whereas low expression may identify those patients who benefit from ICS. This association does not appear to be due simply to reciprocal alterations in type 2 inflammation, as the interaction between our IL-17 signature and treatment was unaffected by adjustments for airway eosinophils or our type 2 airway genomic signature. Thus, our findings suggest that enhanced IL-17 inflammation characterizes a distinct subset of COPD, and that identifying this subgroup may be important for therapeutic decisions.

In COPD, chronic exposure to smoking, microbial insults, and recurrent mucosal injury may all contribute to immune activation with IL-17–producing T cells, supported by innate IL-17–producing cells (17). This likely contributes to ongoing neutrophilic inflammation and macrophage recruitment with subsequent airway remodeling and tissue destruction (48). We found that our IL-17 gene expression signature is associated with increases in airway neutrophils and macrophages, indicating an IL-17 response. It is related to worse clinical outcomes across former and current smokers. These findings provide evidence for the contribution of IL-17 inflammation to COPD pathology despite smoking cessation.

We found that the IL-17 response in COPD is heterogeneous, enhanced in a subgroup. Prior studies found variability in IL-17–related inflammation within COPD (13, 25–30), and our data suggest that this variability is clinically significant. Other studies have identified some characteristics of IL-17–associated inflammation in COPD, including more severe obstruction, emphysema, and lymphoid neogenesis (13, 15). Here we comprehensively investigated the associations between IL-17–driven inflammation and COPD patient characteristics. In addition to an association with increased airway obstruction, we found associations with a novel CT biomarker of functional small airways disease and corticosteroid unresponsiveness. COPD phenotypes are heterogeneous and complex. Thus we hypothesize that multiple overlapping molecular phenotypes underlie the complex clinical phenotypes we observe in chronic airway diseases and that there will be an upper bound to the predictive power of any one biological pathway (33, 49). However, a strength here is that we observe correlations that are reproducible across our transcriptional data sets (for associations with neutrophils and FEV_1_).

We had hypothesized that the IL-17 signature would be associated with increased emphysema, as found in a previous study (13). We evaluated this using the recently developed PRM CT analysis method (46). By matching inspiratory and expiratory scans, PRM improves the ability to distinguish emphysema from functional small airways disease, both of which are associated with low-radiodensity lung regions on expiration (i.e., air trapping). Our IL-17 signature is associated with PRM^fSAD^ but not PRM^emph^ in SPIROMICS. As the participants generally had mild to moderate disease with minimal emphysema, the lack of association with PRM^emph^ is not surprising. The association with PRM^fSAD^ is of interest as the small airways are likely the main site of airway inflammation in COPD, and small airways disease is thought to precede emphysema (50). Studies using PRM have supported these findings. PRM^fSAD^ is associated with more rapid FEV_1_ decline, particularly in mild to moderate disease (47). PRM^fSAD^ is also the greater contributor to radiographic abnormalities in mild to moderate COPD, with both PRM^fSAD^ and PRM^emph^ contributing in severe disease (46). Thus, an association between our IL-17 signature and PRM^fSAD^ in mild to moderate COPD does not preclude an association with emphysema in more severe disease. In fact, it signifies an association with a more severe phenotype among participants with milder airway obstruction and suggests that IL-17–related inflammation may be a pathway on which to intervene to prevent the progression to emphysema and severe airway obstruction.

Our IL-17 signature, when measured at baseline, is associated with a poor lung function response to corticosteroids at 30 months. This corticosteroid responsiveness is not simply due to participants with low IL-17 signature expression exhibiting low neutrophil counts or reciprocally exhibiting high type 2 inflammation. In murine models, Th17 cell–mediated airway inflammation has been shown to be corticosteroid resistant, in contrast to Th2 cell–mediated inflammation (51). Here, for the first time to our knowledge, we show the association between an IL-17 inflammatory signature and corticosteroid unresponsiveness in a longitudinal randomized controlled trial in humans. Many patients with COPD do not respond to corticosteroids, and ICSs are only indicated in exacerbation-prone symptomatic COPD. However, corticosteroids are still used broadly despite possible increases in adverse outcomes such as pneumonia (52). The corticosteroid unresponsiveness finding suggests that a more easily measurable surrogate for our IL-17 signature could serve as a biomarker for therapeutics in COPD. While it may be useful to predict who will not respond to corticosteroids, it may be even more useful to predict who will respond to therapies targeting IL-17 or associated inflammatory pathways as we found in psoriatic lesions.

Our study relied on the airway epithelial gene expression response to IL-17. The airway epithelium is the first line of defense against injury in the lung and a chief target on which IL-17 induces an effect. Other studies have relied on cell counts or immunoreactivity, which are poorly correlated in the human lung (13). Additionally, Th17 cells display a high level of plasticity, and are thus more unstable than Th1 or Th2 cells (53), suggesting that cell numbers may not represent cytokine response. Data are also conflicting on whether IL-17^+^ cell counts are elevated in COPD and related to key pathologic characteristics such as airway neutrophilia (28, 29). We, however, show that IL-17 signature genes are not only upregulated in 2 separate experiments in which HBECs were stimulated with IL-17, but that our signature is associated with increases in airway neutrophils as well. We also show that our signature is decreased in response to IL-17–blocking agents in psoriatic skin lesions but distinct from airway epithelial type 1 and 2 responses, further indicating that we are marking an IL-17–specific epithelial response.

We acknowledge that fitting our IL-17 signature to *CCL20* could have limited its generalizability. However, the signature generalized well in that (a) it was highly correlated with 2 other IL-17 gene signatures (a signature previously studied in asthma [ref. 39] and a signature fit to *SLC26A4*, the most significantly upregulated gene in our IL-17–stimulated human bronchial epithelial culture experiments) and (b) our IL-17 signature was responsive to anti–IL-17 therapy and reflective of clinical response in 2 randomized controlled trials in psoriasis. The advantage of fitting this gene signature to *CCL20* is that it improved its “fit” to a more complex in vivo tissue environment rather than a simple cell culture model. In a COPD patient this complex environment may be further compounded by multiple airway insults (e.g., smoking, microbial colonization, exacerbations, medications) that are not modeled well in culture. By retaining only tightly intercorrelated genes, a well-established method for identifying genes in the same molecular pathway (36, 37), we removed those genes that may be nonspecific to an IL-17 response in vivo.

Our study has some potential limitations. For instance, some analyses were cross-sectional, and those analyses can only show associations, not causality. Our longitudinal analyses were limited by sample size. Thus, while we did find a strong association between our IL-17 signature and lack of response to inhaled steroids over 30 months, an assessment of the predictive power of the signature for corticosteroid responsiveness was quite limited. Furthermore, our definitions of “high” and “low” for the IL-17 signature are highly dependent on the population in which they were developed. Therefore, further studies will be needed to determine whether the signature could be used as a biomarker for steroid unresponsiveness, and to determine the best cutoff for IL-17 high and low. We also were not powered to study the association between the signature and exacerbation rates, which will be important to study in relation to therapeutic response. It was not within the scope of this study to identify the cause of the increased IL-17 response. We do, however, see associations in current and former smokers, suggesting that more than just smoke exposure is playing a role. The contributions of stimuli such as alterations in the microbiome or autoimmunity to enhanced IL-17–related gene expression will require further study. Furthermore, COPD phenotypes are heterogeneous and complex, and thus we hypothesize that multiple overlapping molecular phenotypes underlie the complex clinical phenotypes we observe in chronic airway diseases. Finally, future work will be needed to identify surrogate biomarkers in more easily obtained specimens than airway brushings. This is similar to the approach we took in our asthma studies in which we initially identified a type 2 high asthma molecular phenotype based on airway gene expression, and then expanded this work to identify the best associated biomarkers (periostin, eosinophils, FeNO).

In summary, we show here that a signature of IL-17–associated airway inflammation is upregulated in approximately a third of COPD participants and is associated with distinct inflammatory, physiologic, and clinical features. Our findings suggest that the IL-17 signature defines a molecular COPD phenotype that responds poorly to corticosteroid therapy, and that could instead be the target of emerging therapies that interfere with IL-17 (44, 45, 48).

## Methods

### Transcriptomic data sets

Eight transcriptomic data sets were used for these analyses:

#### UCSF human bronchial epithelial cell culture data set.

Human bronchial epithelial cells (HBECs) obtained from the proximal airways of 6 lung donors rejected for transplant (5 without airway disease, 1 with asthma) were grown to confluence in an air-liquid interface (ALI) culture for 28 days as described previously (54). Some cultures were stimulated with IL-17 (10 ng/ml) for the final 7 days of culture or IFN-γ (10 ng/ml) for the final 24 hours of culture. Matched cultures maintained in media without cytokine over the same time period were used as controls. Cultured cells were then harvested and underwent RNA isolation using the Qiagen miRNeasy kit (Qiagen Inc.) per the manufacturer’s protocol. RNA quality and quantity were assessed using the Agilent 2100 Bioanalyzer (Agilent Technologies) and the NanoDrop Spectrophotometer (Thermo Fisher Scientific). Library preparation and multiplexing were done using the Illumina TruSeq Stranded Total RNA with Ribo-zero Human/Mouse/Rat kit (Illumina Inc.) per the manufacturer’s protocol at the UCSF Sandler Genomics Core Facility. One-hundred-base-pair paired-end sequencing was done on multiplexed samples via the Illumina HiSeq 2500 at the UCSF Genomics Core.

#### Bronchial airway epithelial data set.

Bronchial epithelial brushings obtained from sixth- to eighth-generation bronchi of former and current smokers with a range of lung function (COPD, 85; no COPD, 152) were previously profiled by Affymetrix HG 1.0 ST Arrays (55). Spirometry was done in all participants. Raw microarray files may be downloaded from the Gene Expression Omnibus (GEO; accession GSE37147) (54). Inclusion/exclusion criteria were previously published.

#### Validation HBEC culture data set.

Data were downloaded from GEO (GSE10240). Primary HBECs provided by the Tissue Core Laboratory at the University of Pittsburgh (Pittsburgh, Pennsylvania, USA) or purchased from Cambrex (Lonza) were grown to confluence in ALI, then stimulated apically and basolaterally with media control or IL-17 for 24 hours (3 replicates each) as previously described (43). Isolated RNA was profiled by Affymetrix HG U133A 2.0 Arrays.

#### Asthma data set.

Bronchial airway epithelial (BAE) brushings obtained by bronchoscopy from steroid-naive subjects with mild to moderate asthma (*n* = 62) and control subjects without asthma (*n* = 43) were previously profiled by Affymetrix HG U133 Plus 2.0 Arrays (GEO GSE67472) (24). Inclusion/exclusion criteria for this study were previously published (24). Subjects with asthma were divided into type 2–high and –low subgroups (*n* = 40 and 22, respectively) using a validated standardized mean expression level of *POSTN*, *SERPINB2*, and *CLCA1* (24, 32). IL-17–associated genes were evaluated among those differentially expressed between type 2–high asthma and type 2–low asthma and healthy controls.

#### Ixekizumab psoriasis data set.

Data were downloaded from GEO (GSE31652). Biopsies of psoriatic skin lesions were taken at baseline and after treatment for 2 weeks with ixekizumab (*n* = 6) or placebo (*n* = 4) and previously profiled by Affymetrix HG U133A 2.0 Arrays (44).

#### Brodalumab psoriasis data set.

Data were downloaded from GEO (GSE53552). Biopsies were taken from psoriatic skin lesions and matched nonlesional skin from 25 participants at baseline. The psoriatic lesions were then sampled over 6 weeks after treatment with placebo (*n* = 5) or a dose range of brodalumab (140 mg, *n* = 4; 350 mg, *n* = 8; 700 mg, *n* = 8). All samples were previously profiled by Affymetrix HG U133 Plus 2.0 Arrays (45).

#### Groningen and Leiden Universities Study of Corticosteroids in Obstructive Lung Disease (GLUCOLD) data set.

Endobronchial biopsies from steroid-naive participants with moderate to severe COPD (*n* = 79) were previously obtained by bronchoscopy and profiled by Affymetrix HG 1.0 ST Arrays (GEO GSE36221) (56). Blood collection, sputum induction, and spirometry were done at the first study visit via previously described methods (57). A subset of these participants was randomized to receive 30 months of placebo (*n* = 16) or ICS with or without long-acting β-agonist (salmeterol) (*n* = 33). Inclusion/exclusion criteria were previously published (57).

#### Subpopulations and Intermediate Outcome Measures in COPD Study (SPIROMICS) data set.

A subgroup of participants in the SPIROMICS multicenter observational cohort study underwent research bronchoscopy. RNA was obtained from bronchial epithelial brushings from third- to fourth-generation bronchi of the right or left lower lobe of current and former smokers with mild to moderate COPD (*n* = 47). RNA was used for profiling IL-17–associated gene expression by 2-step, nested-primer quantitative reverse transcription PCR as described previously (32). Primer and probe sequences are listed in Supplemental Table 8.

At least a 20-pack-year smoking history was required for inclusion, and participants were classified as former smokers after 1 year of smoking cessation. Participants were classified as having COPD based on spirometry, performed before and after 4 inhalations each of albuterol (90 μg dose per inhalation) and ipratropium (18 μg dose per inhalation), using the GOLD staging system (58). Full inclusion/exclusion criteria are included in Supplemental Table 7.

Blood collection, sputum induction, and CT scans were done at the first study visit. Sputum induction was performed as previously described (59). Parametric response mapping (PRM) of CT imaging was used to distinguish areas of normal lung (PRM^norm^) from areas of functional small airways disease (PRM^fSAD^) and emphysema (PRM^emph^) as previously described (46, 47). Briefly, PRM is a CT voxel-based imaging biomarker that uses dynamic image registration to spatially align paired inspiratory and expiratory scans. PRM^fSAD^ is defined as areas of lung that are greater than –950 Hounsfield units (HU) on inspiration and less than –856 HU on expiration. PRM^emph^ is defined as areas of lung that are less than –950 HU on inspiration and less than –856 HU on expiration. PRM^norm^ is defined as areas of lung exceeding both thresholds on inspiration and expiration.

### Derivation of gene expression data sets

#### RNA-Seq (HBEC culture data set).

FASTQ files were quality-filtered and aligned to the human genome using STAR and the Ensembl GRCh38 genome build (60, 61). Read counts were normalized and differential expression analyses on matched samples were performed between (a) IL-17–stimulated samples and controls and (b) IFN-γ–stimulated samples and controls using the DESeq2 package in R (62). Differential expression in DESeq2 is carried out using generalized linear models following a negative binomial distribution. Results were trimmed to transcripts indexed in the HUGO Gene Nomenclature Committee (HGNC) database and with an Ensembl gene biotype label of “protein_coding.” Multiple-comparisons corrections were done using false discovery rate (FDR) by the Benjamini-Hochberg method (63).

#### Microarray (BAE, asthma, GLUCOLD, ixekizumab, and brodalumab data sets).

Each microarray data set independently underwent background adjustment (without the use of mismatch probes), quantile normalization, and probe summarization using the RMA algorithm (affy package, Bioconductor, R) (64, 65). Entrez Gene custom chip definition files available for the appropriate microarray for each data set at http://brainarray.mbni.med.umich.edu were used for annotation. Batch effect was minimized using Combat when appropriate (66).

#### Quantitative PCR (SPIROMICS data set).

Data were normalized to the mean of *PPIA*, *RPL13A*, *ACTB*, and *DNAJA1*, determined using the SLqPCR package in R, as described previously (32, 67).

### Derivation of the IL-17 genomic signature

An IL-17 genomic signature specific to bronchial epithelial brushings from smokers was generated using elastic net regression for feature selection in the BAE data set. The 100 genes most upregulated in ALI models after IL-17 stimulation were used as candidate predictor variables (“features”). Genes highly correlated with a representative IL-17 gene, *CCL20*, were selected as features for inclusion in the IL-17 signature using elastic net regression via the glmnet package in R with α = 0.75 and leave-one-out cross-validation (68). Alpha was selected at just below 1 to maximize sparsity (and thus limit feature selection) while allowing for selection of closely correlated genes. *CCL20* and the 10 genes selected by elastic net regression were used for generation of the IL-17 signature. The mean of the zero-centered log_2_-scale gene expression values of these 11 genes was used as the IL-17 airway epithelial signature metric, a previously validated method (33, 39). To confirm that our IL-17 signature was not just specific to *CCL20*, two alternative IL-17 signatures were generated. One was generated using the above procedure with *SLC26A4*, the most upregulated gene following IL-17 stimulation in cell culture also measured in the COPD array data, guiding the elastic net. The other was an IL-17 signature previously studied in asthma and was generated in the same way as previously reported, using the mean value of the zero-centered gene expression of five IL-17–associated genes (39).

For the ixekizumab and brodalumab studies, 4 genes were excluded before derivation of the IL-17 signature metric: 2 genes that were poorly annotated in the microarray platform used (*SAA1*, *SAA2*) and 2 genes that were not expressed above background (*CSF3*, *MTNR1A1*) in these skin biopsies. As there was 100% concordance between the 2 psoriasis studies on genes not expressed above background, we concluded that these genes were poorly expressed in the resident skin cells. We did not, however, change the signature in any way based on knowledge of the genes or relevance in psoriasis. The IL-17 skin signature was thus derived using the mean value of the zero-centered log_2_-scale gene expression values of the remaining 7 genes (*CCL20*, *SLC26A4*, *TNIP3*, *CXCL3*, *CXCL5*, *CXCL6*, and *VNN1*).

### Statistical analyses of the IL-17 genomic signature

All regression analyses were performed using the limma package in R (69). For cross-sectional analyses of the associations between the IL-17 signature and clinical variables (in the BAE, GLUCOLD, and SPIROMICS data sets), linear or logistic regression was used, as appropriate. Analyses were done before and after adjustment for age and smoking status. Race, sex, pack-years, and ICS use were evaluated as potential confounders as well. These variables were, however, left out of the final models, as they were not significantly associated with IL-17 signature expression, and did not significantly alter the relationships between the IL-17 signature and outcomes beyond adjustments for age and smoking status. Data were transformed when necessary for normal distribution. A *P* value less than 0.05 was considered significant. However, multiple-hypothesis testing was done using an FDR when appropriate (63). For the ixekizumab and brodalumab studies, mixed-effects models were used to relate the IL-17 signature (as the outcome variable) to the interaction between treatment and time (fixed effects) across participants (random effect). For longitudinal analyses in GLUCOLD, the interaction between treatment (ICS or placebo) and the baseline IL-17 signature was related to change in FEV_1_ over 30 months. The ICS and ICS plus long-acting β-agonist groups were combined to improve power, as the long-term effects in these groups were comparable. In secondary analyses, the interactions between the IL-17 score and (a) tissue eosinophils, (b) our previously generated metric of type 2 inflammation (the T2S score), or (c) tissue neutrophils were related to change in FEV_1_ over 30 months among those GLUCOLD participants who received ICS (6).

### Clustering

All clustering analyses were performed using Euclidean distance with average linkage as the distance metric. The NbClust package (Bioconductor, R) was used to determine the best participant clustering of the IL-17 signature genes, based on a majority vote of 30 indices that evaluate partitioning (70). NbClust deals with the inherent variability in the many indices available to determine the optimal number of clusters by requiring a consensus vote among these indices on best partitioning. Participants with relatively high expression who clustered separately from the majority of participants were considered “IL-17 high.” Before determination of the best number of partitions, the data sets were first stratified by smoking status given the large effect of smoking on gene expression. Differences among indices in deciding best clustering were generally due to separation of those with IL-17–high expression into 1 or more categories, while those with low expression clustered together. The exceptions were 2 participants in the SPIROMICS data set with low expression that were partitioned into their own groups. Six of 35 participants with relatively high IL-17 gene expression in the BAE data set, 3 of 23 participants in the GLUCOLD data set, and 5 of 18 participants in the SPIROMICS data set were partitioned out as the highest for IL-17 expression. For simplicity, all IL-17 high, including these highest participants, were grouped together.

The IL-17 signature was then discretized into 2 categories, IL-17 high and IL-17 low, using 2 different methods, to use as a categorical predictor for longitudinal analyses in GLUCOLD. Discretization was first based on the best partitioning decided by NbClust, and then alternatively based on the top quartile of signature expression. Ten percent of samples with IL-17 signatures closest to the partition were removed before discretization to diminish overlap.

### Study approval

The included human studies were all approved by the institutional review boards at the institutions involved in sample and data collection. All participants provided written informed consent prior to inclusion in the study.

## Author contributions

SAC and PGW contributed to the conceptualization of the study. LRB, LTZ, and DJE carried out the HBEC culture experiments. SAC, MVDB, IZB, RGB, ERB, RCB, RPB, APC, JLC, MKH, NNH, PSH, RJK, JAK, FJM, WKO, RP, WT, JMW, AS, and PGW were involved in data collection and generation. SAC, MVDB, AF, KI, NB, and PGW contributed to data analysis. All authors participated in critical manuscript writing and editing.

## Supplementary Material

Supplemental data

## Figures and Tables

**Table 3:**
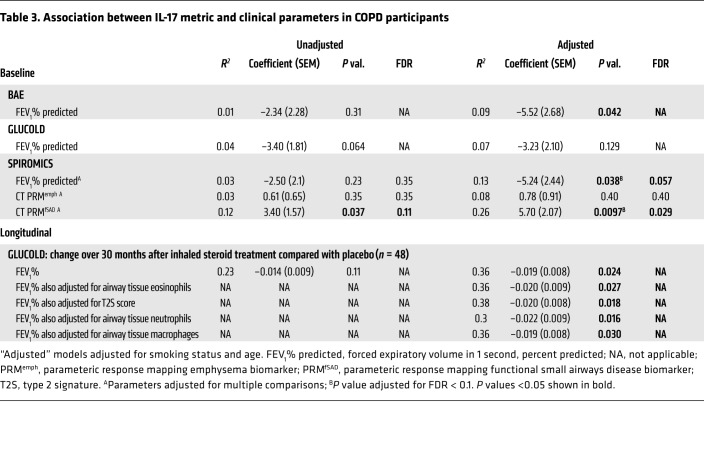
Association between IL-17 metric and clinical parameters in COPD participants

**Table 2:**
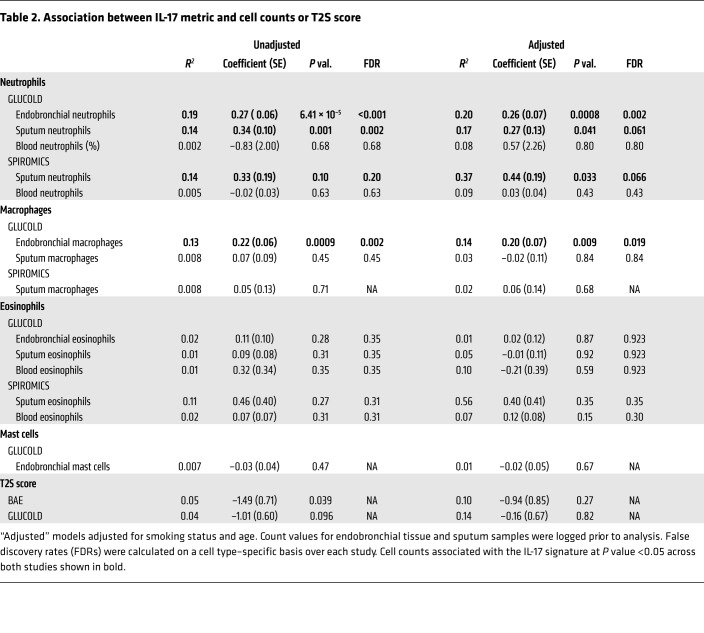
Association between IL-17 metric and cell counts or T2S score

**Table 1:**
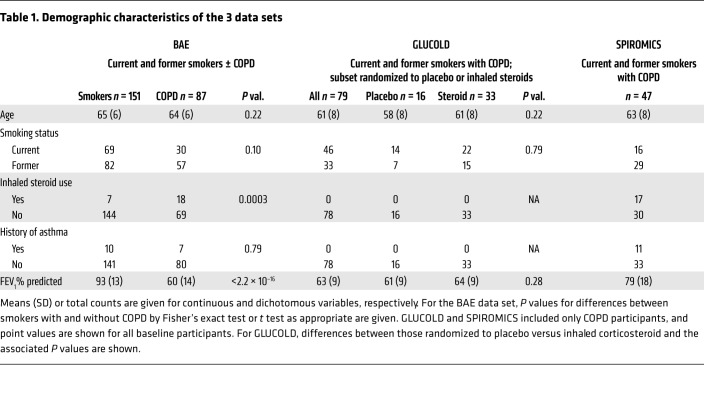
Demographic characteristics of the 3 data sets

## Version 1 11/01/2018

In-Press Preview

## Version 2 11/26/2018

Electronic publication

## Version 3 01/02/2019

Print issue publication
