# Supplementary material for: An airway epithelial IL-17A response signature identifies a steroid-unresponsive COPD patient subgroup
Source: J Clin Invest. 2018 Nov 26;129(1):169–81. doi: 10.1172/JCI121087 (PMC6307967; doi:10.1172/JCI121087)
Supplement: Supplemental data [file jci-129-121087-s106.pdf]

Supplemental Tables and Figures

Table S1. Association between 11 gene IL-17 signature metric and 2 alternative IL-17 signature metrics

	ρ	p-val
**Asthma 5 Gene Signature**		
BAE	0.51	$<2.2*10^{-16}$
**GLUCOLD**	0.49	$5.01*10^{-6}$
***SLC26A4*-Guided Metric**		
BAE	0.97	$<2.2*10^{-16}$
**GLUCOLD**	0.87	$<2.2*10^{-16}$
***100 Gene* Metric**		
BAE	0.72	$<2.2*10^{-16}$
**GLUCOLD**	0.49	$5.01*10^{-6}$

Table S2. Association between IL-17 gene expression and Type 1 inflammation.

Gene Symbol	Log Fold Change	p-value	False Discovery Rate
CCL20	-0.338	0.674	0.911
CSF3	-0.032	0.980	0.999
CXCL3	-0.018	0.965	0.999
CXCL5	-1.258	0.073	0.432
CXCL6	-1.283	$7.95 *10^{-6}$	$2.35 *10^{-4}$
MTNR1A	0.206	0.787	0.951
SAA1	1.064	$5.20 *10^{-6}$	$1.60 *10^{-4}$
SAA2	0.729	0.0001	0.002
SLC26A4	0.325	0.589	0.911
TNIP3	-1.145	0.357	0.911
VNN1	-0.390	0.043	0.305

Log fold gene expression changes, p-values, and false discovery rates for the differential expression of the 11 IL-17 genes in airway epithelial cells exposed to interferon gamma compared to control.

Table S3. Association between IL-17 gene expression and Type 2 inflammation.

Gene Symbol	Log Fold Change	p-value	False Discovery Rate
CCL20	-0.387	0.010	0.041
CSF3	-0.270	0.003	0.018
CXCL3	-0.450	0.002	0.015
CXCL5	-0.122	0.142	0.290
CXCL6	-0.364	0.001	0.006
MTNR1A	-0.005	0.896	0.947
SAA1	NA	NA	NA
SAA2	NA	NA	NA
SLC26A4	0.175	0.451	0.627
TNIP3	-0.088	0.384	0.567
VNN1	-0.631	$8.32 * 10^{-7}$	$2.58 * 10^{-5}$

Log fold gene expression changes, p-values, and false discovery rates for the differential expression of the 11 IL-17 genes in Type 2 high asthmatics when compared with Type 2 low asthmatics and healthy controls in global profiling experiment by microarray. NA=gene not measured well on microarray.

Table S4. Relationship between IL-17 metric and demographics.

	BAE		GLUCOLD		SPIROMICS	
		p-val		p-val		p-val
Age	0.19	**0.004**	0.24	**0.039**	**0.20**	**0.046**
Smoking Status						
Current	-0.42 (0.48)	**$<2.2*10^{-16}$**	0.32 (0.54)	**$2.42*10^{-6}$**	-0.67 (1.18)	**$1.35*10^{-5}$**
Former	0.29 (0.46)		-0.23 (0.42)		0.35 (0.98)	
Inhaled Steroid Use						
Yes	0.02 (0.59)	0.28	NA	NA	0.34 (1.08)	0.25
No	-0.10 (0.59)				0.09 (1.50)	
History of Asthma						
Yes	0.22 (0.37)	0.06	NA	NA	-0.05 (1.60)	0.99
No	-0.02 (0.60)				-0.06 (1.04)	
History of Childhood Asthma						
Yes	NA	NA	NA	NA	-0.17 (1.25)	0.91
No					-0.03 (1.17)	

For continuous variables rho and p-values from Spearman's correlations are shown. For dichotomous variables means and standard deviations (in parentheses) as well as p-values from Fischer's exact test are given.

Table S5. IL-17 Dichotomization by Gene Expression Partitioning.

	Steroid Unresponsive	Steroid Responsive	
IL-17 High	9	4	Positive Predictive Value 69%
IL-17 Low	9	12	Negative Predictive Value 57%
	Sensitivity 50%	Specificity 75%	

Table S6. IL-17 Dichotomization at Upper Quartile.

	Steroid Unresponsive	Steroid Responsive	
IL-17 High	8	1	Positive Predictive Value 89%
IL-17 Low	10	15	Negative Predictive Value 60%
	Sensitivity 44%	Specificity 94%	

Table S7. Inclusion/Exclusion Criteria for the SPIROMICS bronchoscopy sub-study COPD Participants.

**Inclusion Criteria**
Between age 40 and 80
Able to tolerate and willing to undergo study procedures
>20 pack-year history of smoking
Post bronchodilator: FEV1/FVC < 70% and FEV1 > 30% predicted
Able to understand English and/or Spanish
**Exclusion Criteria**
Women only: Cannot be pregnant at baseline or plan to become pregnant during the course of the study
Dementia or other cognitive dysfunction which in the opinion of the investigator would prevent the participant from consenting to the study or completing study procedures
Has plans to leave the area in the next 3 years
Smoking history of > 1 pack-year but <20 pack-years
Has a BMI > 40 kg/m2 at baseline exam
Prior significant difficulties with pulmonary function testing
Hypersensitivity to or intolerance of albuterol sulfate or ipratropium bromide or propellants or excipients of the inhalers
Non-COPD obstructive lung disease (various bronchiolitides, sarcoid, LAM, histiocytosis X) or parenchymal lung disease, pulmonary vascular disease, pleural disease, severe kyphoscoliosis, neuromuscular weakness, or other conditions, including clinically significant cardiovascular and pulmonary disease, that, in the opinion of the investigator, limit the interpretability of the pulmonary function measures.
History of Interstitial lung disease
Current diagnosis of asthma
History of Lung volume reduction surgery or lung resection
History of lung or other organ transplant
History of endobronchial valve therapy
History of large thoracic metal implants (e.g., AICD and/or pacemaker) that in the opinion of the investigator limit the interpretability of CT scans
Currently taking >=10mg a day/20mg every other day of prednisone or equivalent systemic corticosteroid
Currently taking any immunosuppressive agent
Current illicit substance abuse, excluding marijuana
History of or current use of IV Ritalin
History of or current use of heroin
History of illegal IV drug use within the last 10 years or more than 5 instances of illegal IV drug use ever
Known HIV/AIDS infection
History of lung cancer or any cancer that spread to multiple locations in the body
History of or current exposure to chemotherapy or radiation treatments that, in the opinion of the investigator, limits the interpretability of the pulmonary function measures.
Diagnosis of unstable cardiovascular disease including myocardial infarction in the past 6 weeks, uncontrolled congestive heart failure or arrhythmia.
Bronchoscopy substudy only: Pao2/Sao2 that qualifies them for supplementary oxygen at rest (PaO2< 60 orSaO2<88%)
Bronchoscopy substudy only: History of cardiac disease or other comorbid condition severe enough to significantly increase risks based on investigator discretion.
Bronchoscopy substudy only: Use of anticoagulation (patients on warfarin or clopidogrel will be excluded, patients on aspirin alone can be studied even with concurrent use)

Table S8. RT-PCR primers.

Probes used a 5' FAM fluorescent probe with a 3' BHQ quencher.

Gene	Type	Sequence
CCL20	outer-forward	GGCTGTGACATCAATGCTATCATC
	outer-reverse	GTCCAGTGAGGCACAAATTAGATAAG
	inner-forward	TCTGGAATGGAATTGGACATAGCCCAAG
	inner-reverse	ACCCTCCATGATGTGCAAGTGAAACC
	probe	CCAACCCCAGCAAGGTTCTTTCTG
CSF3	outer-forward	CCTTCCCTGCATTTCTGAGTTT
	outer-reverse	TGATACTCTCAAGGGTCCCAGGT
	inner-forward	CCTGGACTGGGAGGTAGATAGGT
	inner-reverse	CCCTGGCTCTGCAATGGGCACT
	probe	GGCTCACAGCGGCTCATC
CXCL3	outer-forward	CTGCAGGGAATTCACCTCAAG
	outer-reverse	TCTCTCCTGTCAGTTGGTGCTC
	inner-forward	AGCCACACTCAAGAATGGGAA
	inner-reverse	TTTTCGATGATTTTCTGAACCATG
	probe	AAGCTTGTCTCAACCCCGCATCCC
CXCL5	outer-forward	CTCCTTGTGCGCGCTGTT
	outer-reverse	GTTCTTCAGGGAGGCTACCACTT
	inner-forward	AGAGCTGCGTTGCGTTTGT
	inner-reverse	GGCGAACACTTGCAGATTACTG
	probe	TACAGACCACGCAGGGAGTTCATCCC
CXCL6	outer-forward	TACTTTGAAGAGTGTGGGGGAAAG
	outer-reverse	GCCTTTTCGGTAAGACTTTAAGGA
	inner-forward	GCCTACGCTTCTCCCTGAAG
	inner-reverse	AACCAGTGATTCTTTGCTCACAA
	probe	TTGAACCCTTTGGCAATTGACCA
MTNR1A	outer-forward	ATATTTAACAACGGGTGGAACCTG
	outer-reverse	ACTTGAGACTGTGGCAGATGTAGC
	inner-forward	GGAACCTGGGCTATCTGCAC
	inner-reverse	TGAATATGGAGCCGATGACG
	probe	TCAGGCCCATCAGGAACCCACTG
SAA1	outer-forward	ATCGGCTCAGACAAATACTTCCAT
	outer-reverse	AGCAGAGTGAAGAGGAAGCTCAGT
	inner-forward	AAGTGATCAGCGATGCCAGA
	inner-reverse	TGATCAGCCAGCGAGTCCT
	probe	CCGCACCATGGCCAAAGAATCTCT
SAA2	outer-forward	GCCTTTGGAACCTGTGCTAAGA
	outer-reverse	AGAGAATGGAGACACCACACTGAG
	inner-forward	CCTGTGCTAAGAGGCATGGA
	inner-reverse	CCACACTGAGCCTTCAGACC
	probe	CCATCCACATGCTGAGGGAGCTCA
SLC26A4	outer-forward	TCAAGACATATCTCAGTTGGACCTTT
	outer-reverse	ACAGTTCCATTGCTGCTGGATAC
	inner-forward	GGTGAGTTTAATGGTGGGATCTG
	inner-reverse	TGCTGCTGGATACGAGAAAGTG
	probe	TGAGCATGGCCCCCGACGA
TNIP3	outer-forward	GTAGCAGAGCTGAAGACGAAACTG
	outer-reverse	GTCCTCTCTCTGCCTGTCGTC
	inner-forward	GCGGAAAGATTCCTCAGCAC
	inner-reverse	TGCCTGTCGTCCTTTCTCTG
	probe	CTGATGCGGATCCTTCTCCCGC
VNN1	outer-forward	CCCAATTCTTCAAGAGCATTTCAT
	outer-reverse	GATGAGAGCGCTTCTATACTGCTG
	inner-forward	ACTCCTCCTCTCGCAACTGG
	inner-reverse	CTGCTGGCATAGGAAGTCCA
	probe	TTCCCACCCATCCCATTCTGCAG

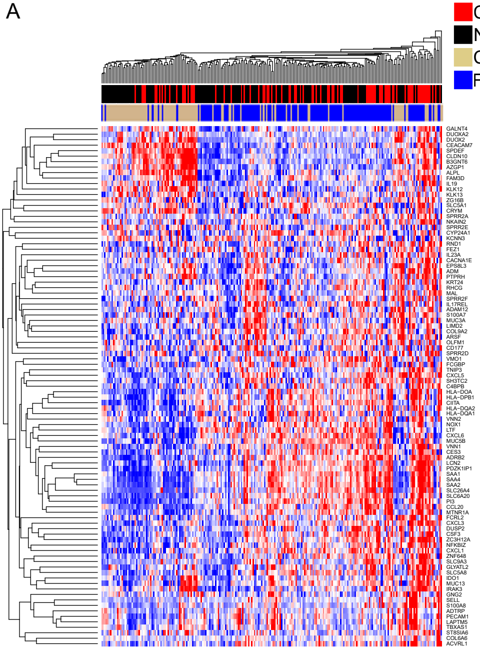

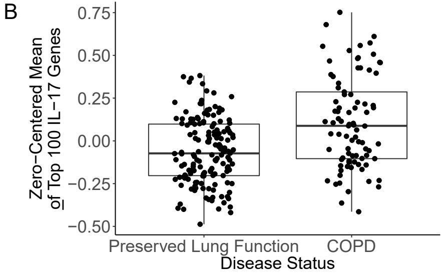

Figure S1. The top 100 genes increased in cultured BECs in response to IL-17 evaluated in the BAE dataset (n=237). (A) Hierarchical clustering of the top genes induced by IL-17 relative to vehicle control (rows) across participants (columns) in the BAE dataset. Five genes were excluded as they were not measured or were in low abundance in the BEC microarray dataset. Blue indicates low and red indicates high relative gene expression. Smokers with and without COPD are indicated by red and black and smoking status by blue and tan in the above color bars, respectively. (B) The mean of the zero-centered log2 expression of these 95 genes is increased in smokers with COPD (0.11±0.27) compared to those without COPD (-0.06±0.19, $p<0.001$ by Wilcoxon Rank Sum Test).

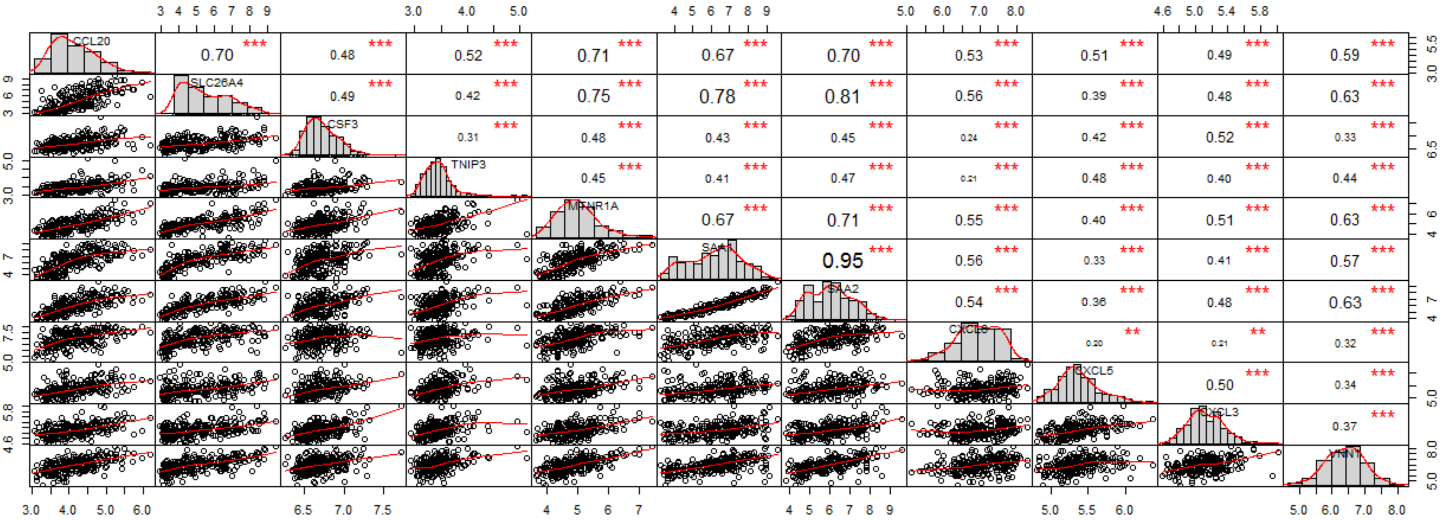

Figure S2. Correlation Matrix of the 11 genes included in the IL-17 signature. The name and distribution of each gene is shown along the diagonal. Below the diagonal are bivariate scatter plots showing the correlation for each gene pair with a line of fit. Above the diagonal are the spearman correlation rho values for each gene pair. Significance levels are indicated by ***: p=0, **: p<0.001.

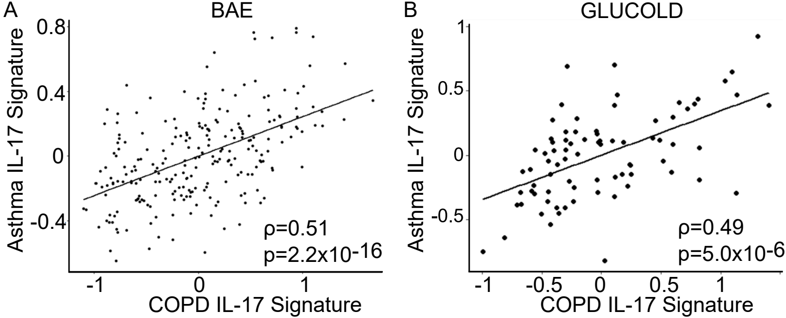

Figure S3. Correlation between the IL-17 signature and a 5 gene IL-17 signature previously evaluated in asthma in (A) the BAE dataset and (B) the GLUCOLD dataset. ρ and p-values shown for spearman correlation.

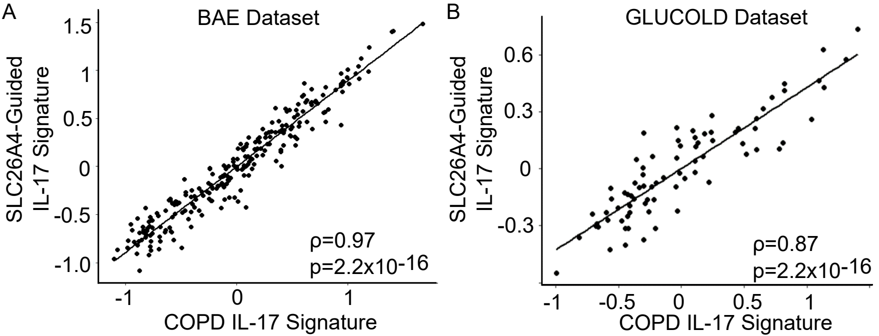

Figure S4. Correlation between the IL-17 signature and an additional IL-17 gene signature generated from the 16 genes selected using an elastic net guided by the gene *SLC26A4* in (A) the BAE dataset and (B) GLUCOLD. ρ and p-values shown for spearman correlation.

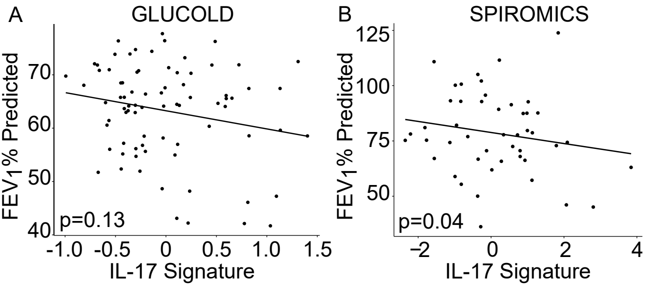

Figure S5. The IL-17 signature is associated with decreasing FEV1% predicted amongst COPD participants. (A) Only a trend in GLUCOLD ($p=0.13$), (B) but significant in SPIROMICS ($p=0.04$). P-values for linear models adjusted for age and smoking status.

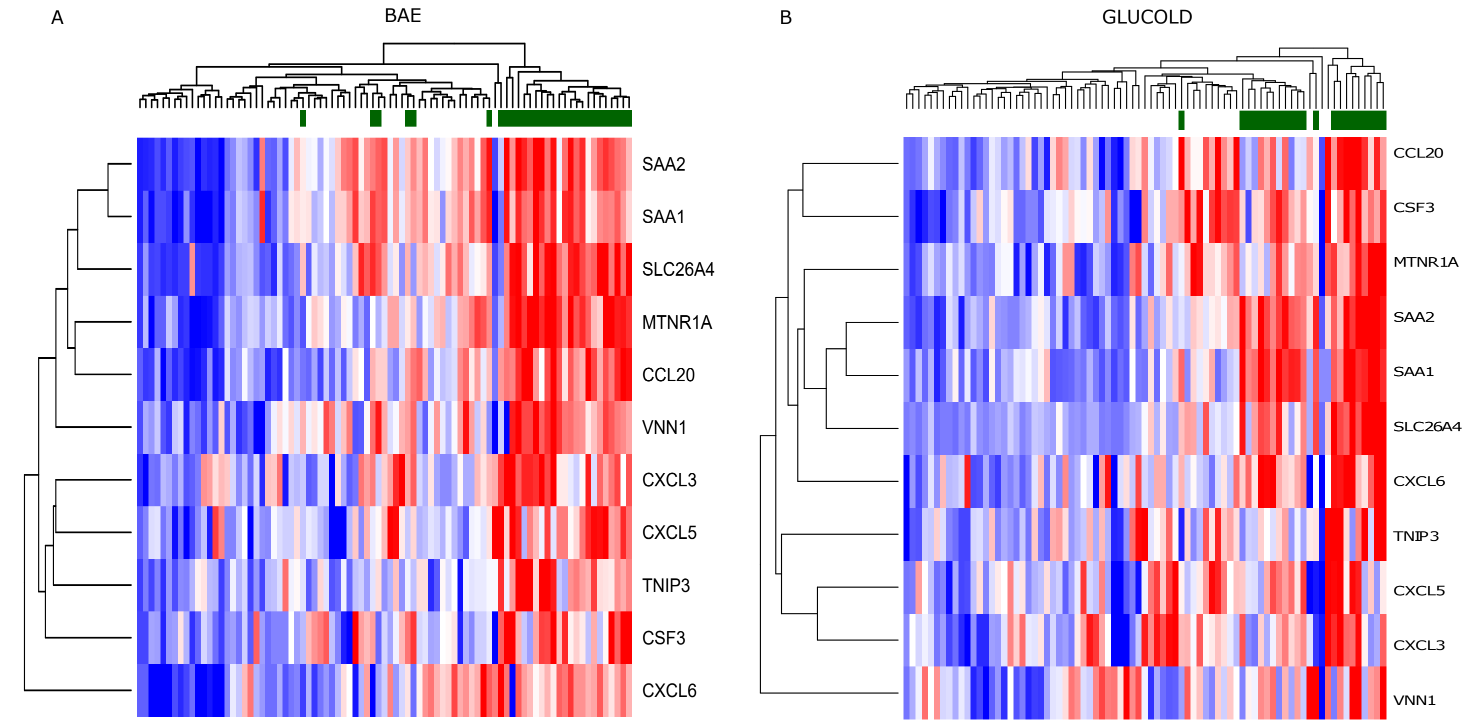

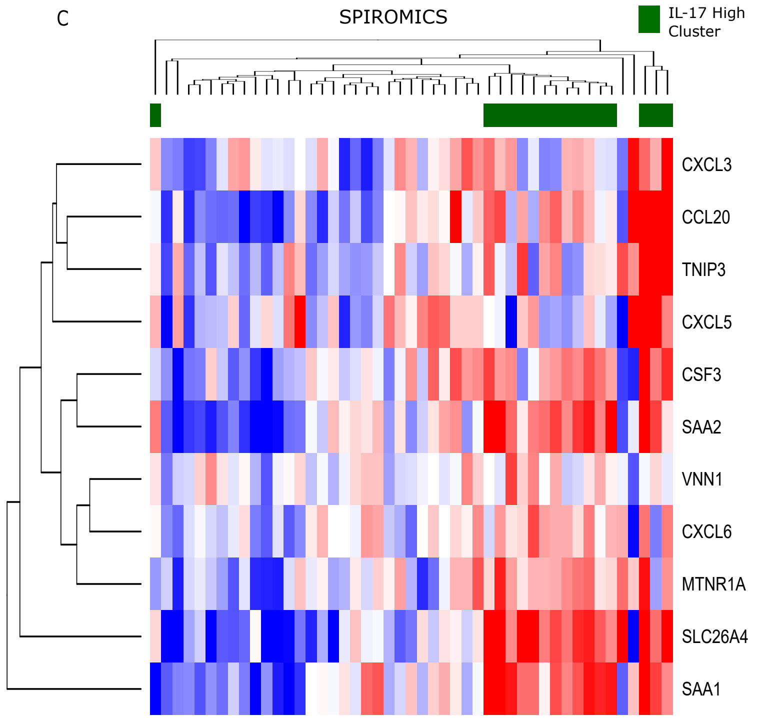

Figure S6. Cluster analyses amongst COPD participants across the three datasets. Hierarchical clustering by Euclidean distance with average linkage of the 11 genes in the IL-17 signature (rows) across COPD participants (rows) in the (A) BAE dataset (n=237), (B) GLUCOLD dataset (n=79), and (C) SPIROMICS dataset (n=47). Blue and red indicate low and high relative gene expr ession, respectively. “IL-17 high” participants are indicated by green in the color bars above the heatmaps.

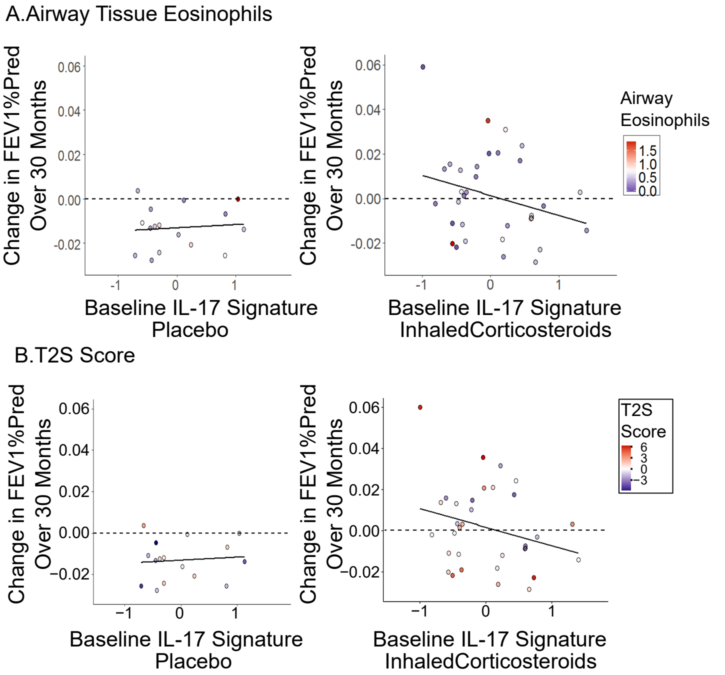

Figure S7. The interaction between the IL-17 signature, Airway Eosinophils or T2S score, and ICS response in GLUCOLD. Increasing IL-17 signature expression was associated with decreasing FEV1 percent change from baseline in GLUCOLD participants on ICS ± long acting beta agonist (n=33) compared to placebo (n=16) at 30 months. This association was not affected by airway eosinophil counts or the Type 2 Signature (T2S) score (p=0.027 and 0.018 for the interaction between the IL-17 signature and treatment following adjustment for airway tissue eosinophils or T2S score, respectively, in addition to age and smoking) as shown by the color gradient for (A) airway tissue eosinophils and (B) T2S score.

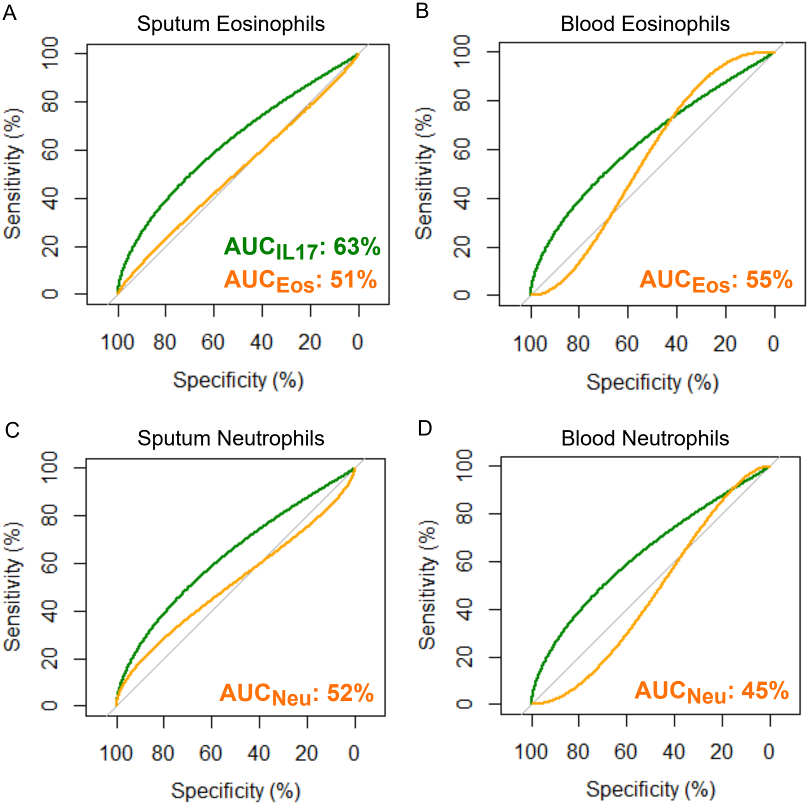

Figure S8. ROC Curves of Steroid Responsiveness for the IL-17 Signature and Cell Count Biomarkers in GLUCOLD. Smoothed Receiver Operator Characteristic (ROC) curves for the IL-17 gene signature in green and cell counts in orange (A. log sputum eosinophils, B. blood eosinophils, C. log sputum neutrophils, D. blood neutrophils) to predict an improvement in FEV1 after 30 weeks of ICS. Area Under the Curve (AUC) shown in green for the IL-17 signature and orange for cell counts. The differences between the IL-17 curve and cell count curves were not statistically significant by bootstrapping (pROC package, R).

Supplemental Acknowledgements: The authors thank the SPIROMICS and GLUCOLD participants and participating physicians, investigators and staff for making this research possible. More information about the study and how to access SPIROMICS data is at www.spiromics.org. We would like to acknowledge the following current and former investigators of the SPIROMICS sites and reading centers: Neil E Alexis, PhD; Wayne H Anderson, PhD; R Graham Barr, MD, DrPH; Eugene R Bleecker, MD; Richard C Boucher, MD; Russell P Bowler, MD, PhD; Elizabeth E Carretta, MPH; Stephanie A Christenson, MD; Alejandro P Comellas, MD; Christopher B Cooper, MD, PhD; David J Couper, PhD; Gerard J Criner, MD; Ronald G Crystal, MD; Jeffrey L Curtis, MD; Claire M Doerschuk, MD; Mark T Dransfield, MD; Christine M Freeman, PhD; MeiLan K Han, MD, MS; Nadia N Hansel, MD, MPH; Annette T Hastie, PhD; Eric A Hoffman, PhD; Robert J Kaner, MD; Richard E Kanner, MD; Eric C Kleerup, MD; Jerry A Krishnan, MD, PhD; Lisa M LaVange, PhD; Stephen C Lazarus, MD; Fernando J Martinez, MD, MS; Deborah A Meyers, PhD; Wendy C Moore, MD; John D Newell Jr, MD; Laura Paulin, MD, MHS; Stephen Peters, MD, PhD; Elizabeth C Oelsner, MD, MPH; Wanda K O'Neal, PhD; Victor E Ortega, MD, PhD; Robert Paine, III, MD; Nirupama Putcha, MD, MHS; Stephen I. Rennard, MD; Donald P Tashkin, MD; Mary Beth Scholand, MD; J Michael Wells, MD; Robert A Wise, MD; and Prescott G Woodruff, MD, MPH. The project officers from the Lung Division of the National Heart, Lung, and Blood Institute were Lisa Postow, PhD, and Thomas Croxton, PhD, MD.
